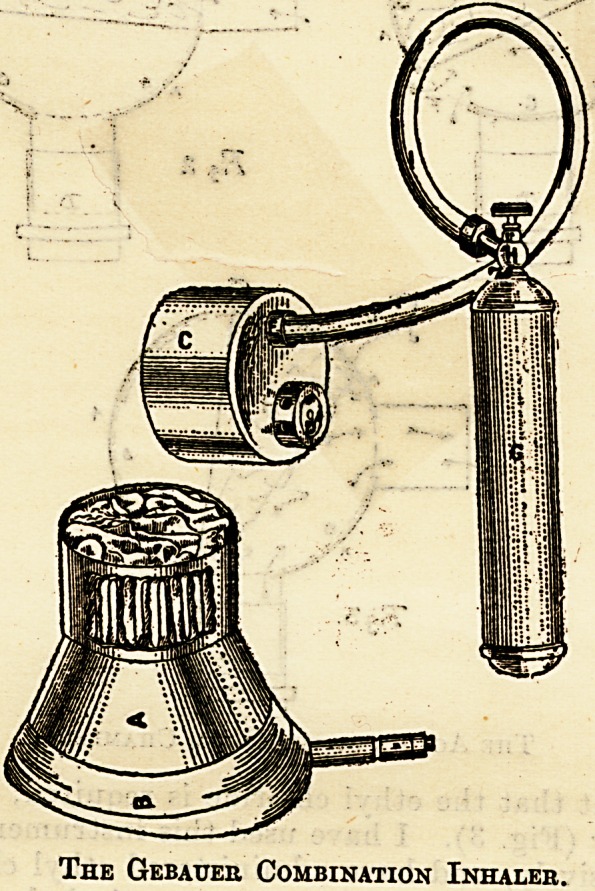# Ethyl Chloride Anæsthesia: Practical Points in Its Administration

**Published:** 1906-10-20

**Authors:** 

**Affiliations:** Anæsthetist to the Central London Hospital for the Throat, Nose, and Ear; formerly Civil Surgeon, Netley Hospital; Medical Officer, Ashanti Field Force


### New Anæsthetic Methods.

### ETHYL CHLORIDE ANÆSTHESIA: PRACTICAL POINTS IN ITS ADMINISTRATION

By Dr. Lauzun-Brown, L.R.C.P., L.R.C.S. (Edin.), Anæsthetist to the Central London Hospital for the Throat, Nose, and Ear; formerly Civil Surgeon, Netley Hospital; Medical Officer, Ashanti Field Force.

### The Search for Anæsthesia.

The search for some material antidote for pain is as old as humanity. Local applications were the first to be practised to dull pain, for diagnosis was easy and the treatment almost specific. Pain was stopped by numbing by the application of ice or snow, and sometimes by tying the limb. The knowledge that certain gases could produce anæsthesia was known long before the power of the Grotto del Cane had declared itself. The Jews gave strong drink (alcohol) to him that was ready to perish, and the Grecian Nepenthe has now become a catchword in poetry. The medieval lore represented by Arabic pharmacy came down continuously till modern times, receiving but few accessions from the schools of the Middle Ages till the coming of the chemical doctors of the seventeenth century.

### Laughing Gas.

Anæthesia as we now understand it is an instance of the growth of knowledge from the smallest grain. Humphrey Davy was the first to hit upon a modern gaseous agent for anæsthesia. As a surgeon's apprentice at Bristol he discovered (in 1800) nitrous oxide or "laughing gas," and pointed out how it might be possible that its deadening powers might be used somewhat in surgery. The medical profession were not then so conscious of their necessities as to grasp at the suggestion. So it was used more as a plaything than otherwise, and thus it came that an American dentist (Mr. Horace Wells), seeing its properties displayed for amusement by an itinerant lecturer (a Mr. Colton) who happened to visit Hertford, Connecticut, to "make the unskilful laugh" with the "gassy frolics," with aptitude and promptness purely American turned the plaything to professional account and made money out of it.

### Ether.

Faraday pointed out in 1818 in the Transactions of the Royal Society that ether vapour mixed with air could produce similar effects to nitrous oxide, but a statement as to its effects on the pulse prevented its general use in this country. And thus again it was left to T. G. Morton, a dentist, and Dr. Charles Jackson, who used pure ether as an anæsthetic in 1846, to demonstrate that ether insensibility was not dangerous to life. The knowledge thus gained in America was warmly acknowledged in this country, though the apparatus and means employed were of the rudest type.

### Chloroform.

Chloroform had been discovered by three workers —in America (Guthrie), France (Soubeiran), and Germany (Liebig) (1831-1832), but not for twenty years were its physiological effects determined. Simpson, tired of the squalid application of sulphuric ether, sought a scientific answer to that most original and rare of all things in science, a scientific question conscientiously and scientifically propounded, "Can an agent be discovered to produce unconsciousness while allowing the free and healthy play of the natural functions?" Chloric ether had been used successfully by Jacob Bigelow of Boston as an anæsthetic, and it was Mr. Waldie, of Linlithgowshire, who suggested to Simpson that the anæsthetic property in that mixture was chloroform or, as it was then called, the perchloride of formyle. Simpson got Messrs. Duncan and Flockhart, chemists in Edinburgh, to make some. The hint led, after much experimental labour, to the famous 4th of November, 1847, when Simpson, Keith, and Duncan had proved to more than their satisfaction the potency of chloroform—a proof within a week made public property. Simpson's work passed through its fiery ordeal of criticism and commission, and now one can say of him what Augustus said of Cicero, "Yes, a great man—a very great man."

### The Search Continued.

The search for other anæsthetics has been continued, and many have been prepared and placed on trial. Some were designed to abolish pain locally, as in the case of cocaine, eucaine, stovaine, and others of that kind, all of which have their uses and their limitations. Among the more important of recent introductions the chloride of ethyl has played a very important part. At first it was employed as a numbing agent—its great volatility rendering it capable of freezing that part of the body upon which it could be sprayed. It was roughly prepared, was not free from impurities, nor, indeed, was it intended to be employed as a general anæsthetic. It was originally used as a toy in France, Germany, and other countries, where it has been manufactured for a long time as a vehicle for spraying scents. The "maffickers" of these countries used this scented ethyl chloride for spraying their friends and acquaintances on gala days. It came to be employed in combination with ethyl bromide and chloride of methyl, a combination described by the trade name of Somnoform. Somnoform had a long trial as a useful anæsthetic in cases where brief anæsthesia was needed. It has fallen into disuse largely because it has been displaced by the cheaper and less complicated and equally useful anæsthetic "pure ethyl chloride." For a long time, however, and with great justice, it should be said Somnoform served a very useful purpose and proved a distinct gain in the field of anæsthesia. Probably the presence of the chloride of methyl in the combination had also some effect in leading to its abandonment as an anæsthetic agent, but the main cause was its decomposition. When a bottle had once been opened it rapidly became decomposed, and so was unsuitable for general practitioners' work.

### Pure Ethyl Chloride.

A fear arose that a similar fate would overtake and lead to the abolition of ethyl chloride as an anæsthetic. In the early days of its administration as a general anæsthetic its powers and properties were unknown; it was badly and clumsily administered, as was the case with sulphuric ether in its early days. All sorts of contrivances, pocket handkerchiefs, cones, towels, and open masks were used, but the volatility of the vapour of ethyl chloride rendered these methods either too costly or too dangerous from the point of view of the patient. Anæsthetists gradually became familiarised with the properties of the ethyl chloride, and suitable appliances were constructed for its administration. Country practitioners who had an ether apparatus bored a hole in the bag-piece, and spraying the ethyl chloride through this into the bag, administered it in that way. This they found answered admirably, except that the india-rubber bag did not profit much by spraying ethyl chloride into it, the ethyl chloride having a tendency to corrode the rubber.

### The Various Inhalers Described.

It is our intention to place before practitioners the appliances used by various skilled anæsthetists whose great experience, long practice, and thorough familiarity with the drug and its capabilities entitle their views to respect. One of the first inhalers to be made on what might be called scientific principles was designed by Dr. George,

Dr. George's Inhaler for Ethyl Chloride.

Senior Anæsthetist to the Central London Throat and Nose Hospital in Gray's Inn Road, where this anæsthetic is largely employed, and where the methods of its administration can be seen on operation days. George's inhaler consists of a face-piece, a bag resembling an ether bag, and a ball-shaped chamber with an aperture above that can be closed at will. The ball is divided by a septum of muslin or some light material into two chambers, anterior and posterior. Into the aperture the quantity of ethyl chloride required is sprayed

The Construction of the Sphere.

from the bottle, the aperture closes automatically, and the administration is commenced. The point is that the ethyl chloride does not come in contact with the rubber, and that it is delivered to the patient as "pure ethyl chloride." The delivery tube in this apparatus is of the same bore as the ordinary ether apparatus, and thus a sudden inrush of ethyl chloride vapour is prevented, with the result that when using George's inhaler very little, if any, chance of laryngeal spasm arises. It is a high-class inhaler, and Dr. George is to be congratulated on having appreciated the necessities of this anæsthetic and for designing an appliance so much in accordance with the requirements of the agent and the comfort of the patient.

An advance on this inhaler is that supplied by Hedley and Co., of Leytonstone. Instead of a ball-shaped chamber, as in Dr. George's, Hedley uses a drum-shaped receptacle. Two pieces fit into each other and are held together by a screw on which they can revolve. One of the pieces carries the

Hedley and Co.'s Drum Chamber Inhaler for Ethyl Chloride.

receiving chamber. A piece of lint is placed in it, on which the ethyl chloride is sprayed through the aperture. The anæsthetic cannot be inhaled by the patient who is breathing pure air meantime until it is pulled forward by means of a lever. All communication with the external air is then shut off, and the patient breathes into the bag. The patient, while the face-piece is close up to the face, breathes with this apparatus pure air (Fig. 1) up to the

The Action of the Drum Chamber.

instant that the ethyl chloride is required, but no longer (Fig. 3). I have used this instrument very extensively, and have administered ethyl chloride considerably over 500 times consecutively by means of this inhaler, and have closely compared its advantages with others which I have had the opportunity of using also extensively. The results have been most gratifying, and the operating surgeons have expressed to me, without knowing anything about the instrument, its superiority over some of the others which were in use. It is the only instrument where a mixture of air and ethyl chloride can be given and regulated if required (Fig. 2).

The third form of ethyl chloride inhaler is one which has been designed by my colleague, Dr. Beresford Kingford. Its simplicity is its great recommendation. It consists of two pieces (no intermediate chamber), a bag, and a mouthpiece. The supporting work of the bag has a notch on one side. A corresponding notch is made on the framework of the face-piece. When these two are fitted an aperture is formed, into which the ethyl chloride can be sprayed, either with or without the intervention of a piece of lint. This aperture can be closed by twisting either the bag or the face-piece. It is a very useful apparatus in Dr. Kingsford's hands. The bag has the wide-bore outlet resembling in that respect Dr. Hewitt's ether inhaler. In some cases the anæsthesia, particularly in children crying and screaming, is very sudden, and has to be watched with much care.

An improvement on this inhaler has been added by Dr. Kingsford whereby the dose can be regulated. A small rubber tube with a stop-cock is attached to the lower end of the bag, and the graduated ethyl-chloride tubes supplied by the manufacturers can be inserted and the dose regulated by the stop-cock. The action of the ethyl chloride on the rubber of the tube necessitates the

Dr. Beresford Kingsford's Ethyl Chloride Inhaler.

frequent changing of the end piece as the rubber loses its elasticity.

Dr. Kingsford's Adaptation.

Messrs. Duncan and Flockhart furnish an inhaler employed by Dr. Luke, the anæsthetist at Edin-

Dr. Luke's Inhaler (Duncan Flockhart and Co.)

burgh Royal Infirmary. It is a very convenient apparatus consisting of a bag, an outlet tube, and a face-piece; the outlet tube is supplied with a small pipe through which the ethyl chloride can be sprayed into the bag, and the tube is closed by means of a small metallic ball. I am particularly impressed with this appliance, and have administered ethyl chloride with it in many hundreds of cases. It is simple in structure, and its bore is narrow. It lacks the intervening chamber which many anæsthetists consider a necessity. The face-piece of this instrument is an excellent celluloid structure, through which the tongue and mouth can be constantly observed, and to which the rubber of the face-piece is securely fastened by means of convenient buttons, thus preventing the unpleasant accident which so frequently happens in a large out-patient department like that of the Central London Hospital, where the rubber slips off the face-piece.

On similar lines to that of Dr. Kingsford is the inhaler manufactured by Wyley's, Limited, Coventry (Coventry House, South Place, Finsbury, London, E.C.). The design is in all respects similar, except that instead of the notches there is an aperture with a tube which conducts the ethyl chloride spray into the bag. This aperture cannot be closed. The structure and workmanship of this inhaler are certainly of a very high standard. It is simple in design and adaptable to Hewitt's inhaler. It has no angles, valves, or loose parts. The face-piece is excellently made, strong and durable, and perfectly safe.

Maw's inhaler is similar in principle to Dr. Luke's, only that the inlet tube is at the side and

Maw's Inhaler.

the ethyl chloride is received on a piece of sponge ingeniously suspended in the centre of the bag. The bag itself is ribbed and uncollapsible. Many other types of inhalers, more or less effective, may be obtained from manufacturers. A curious American instrument is the Gebauer combination-inhaler manufactured at Cleveland. It consists of a metal cone-shaped face-piece with a pneumatic air-pad and a removable top, provided with an exhaling valve and a tube connection. The

The Gebauer Combination Inhaler.

valve is so constructed as to permit a proper quantity of air to enter the inhaler with every inhalation. It also allows the patient to exhale freely. The inhaler is attached to the ethyl-chloride tube by a rubber pipe, and a screw valve regulates the supply of the vapour. In this inhaler the gas is used without any rubber bags. This is a singularly simple, economical, and effective way of administering ethyl chloride.

*(To be continued.)*

## Figures and Tables

**Figure f1:**
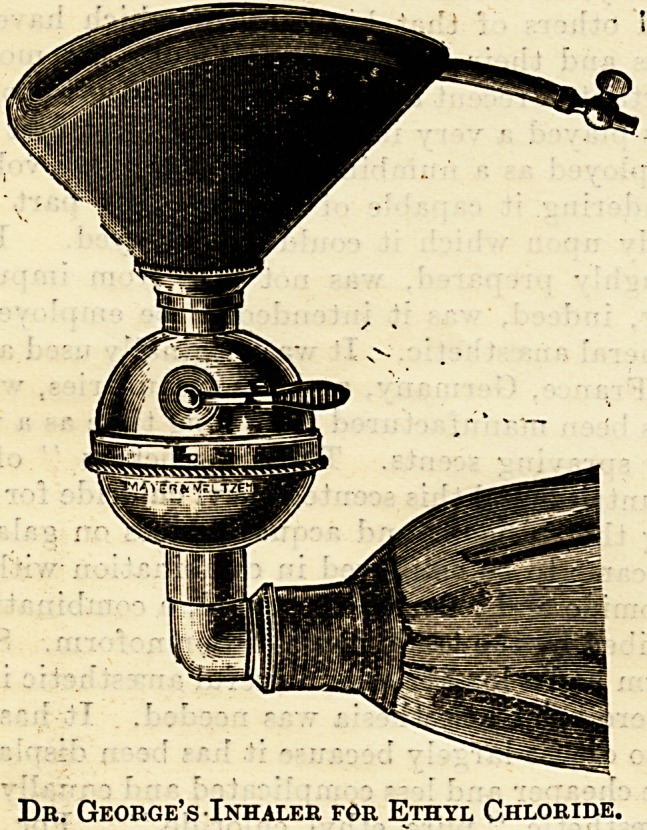


**Figure f2:**
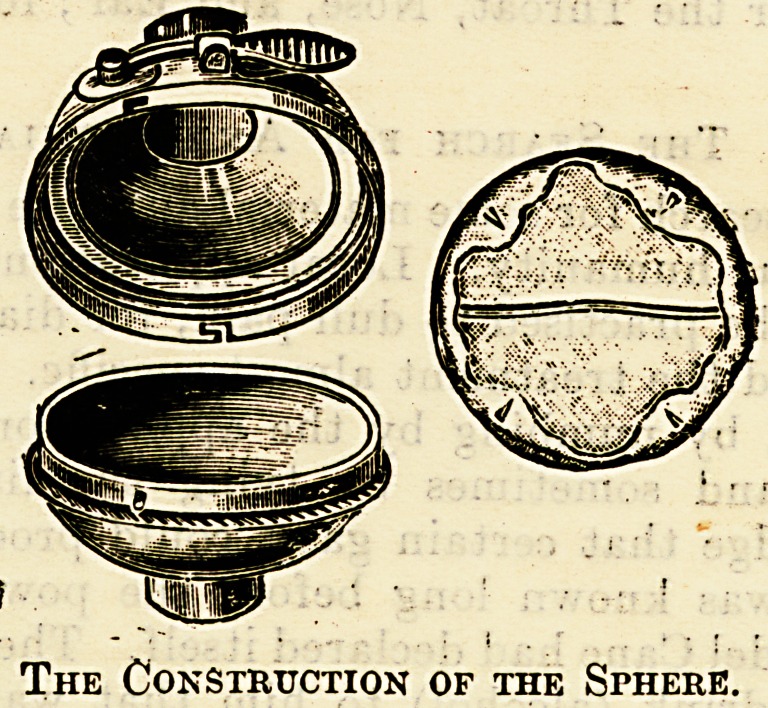


**Figure f3:**
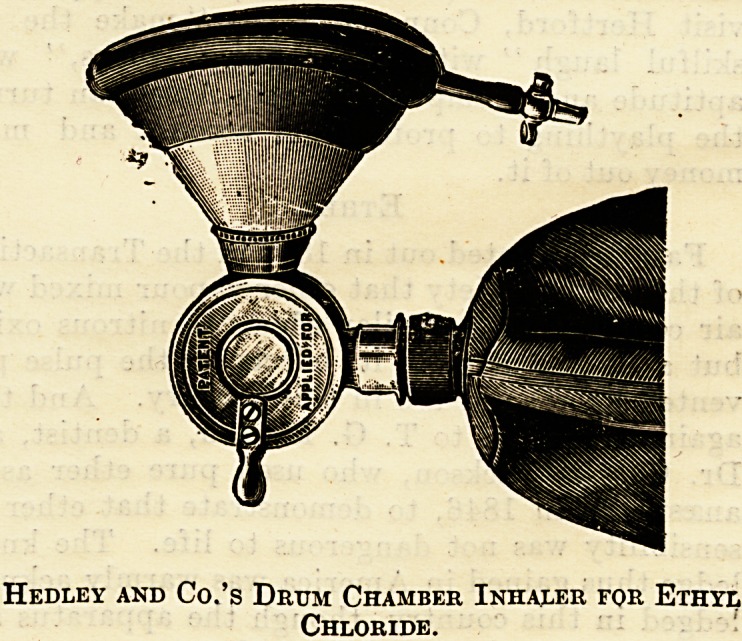


**Fig 1. Fig 2 Fig 3. f4:**
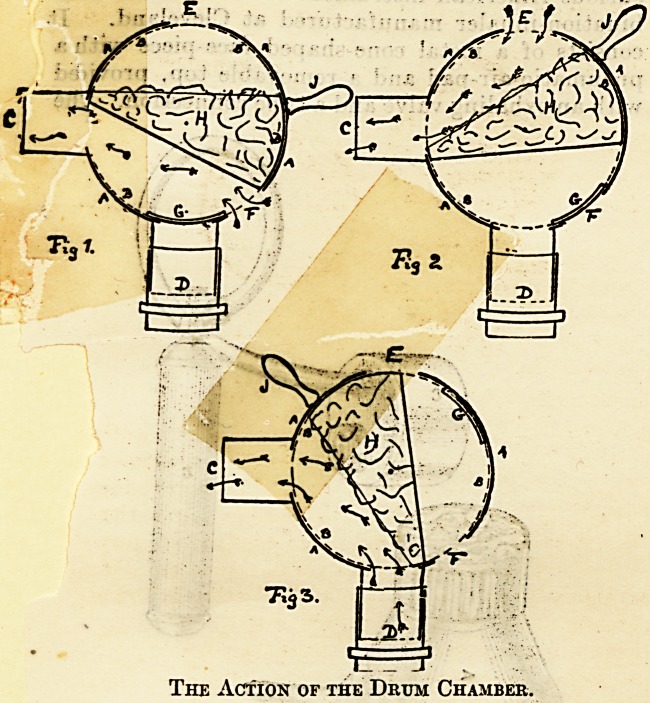


**Figure f5:**
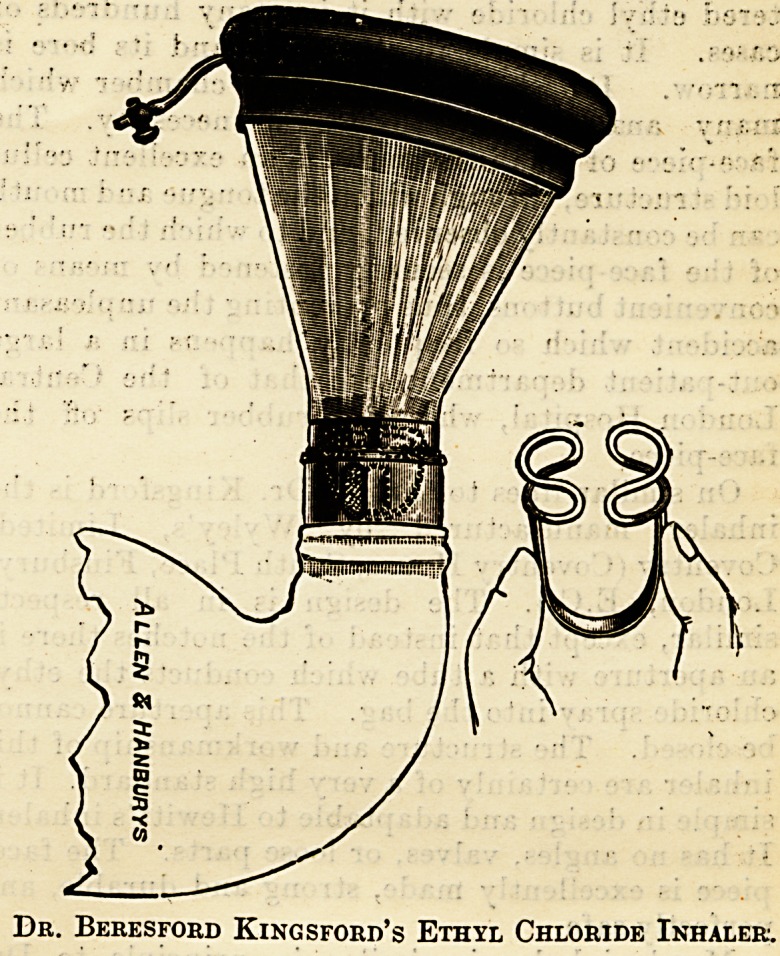


**Figure f6:**
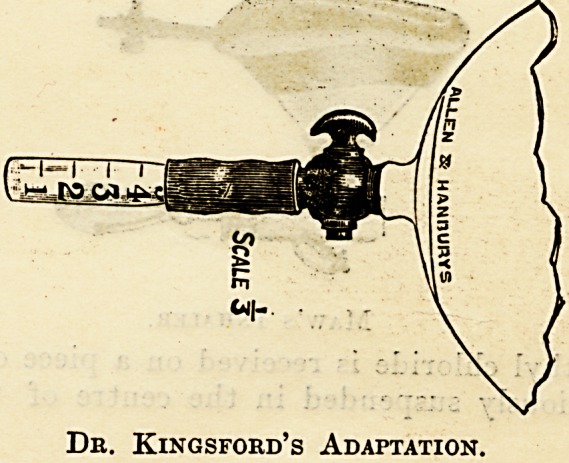


**Figure f7:**
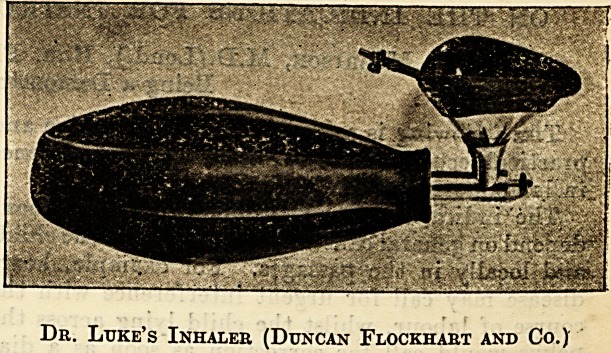


**Figure f8:**
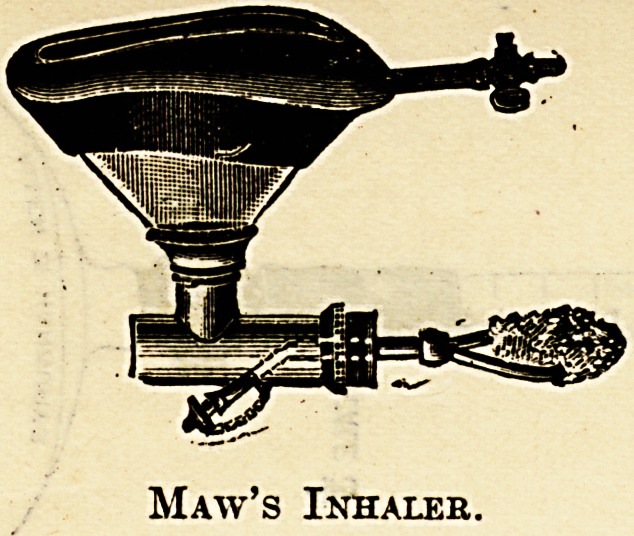


**Figure f9:**